# Caso 2/2020 – Origem Anômala da Artéria Coronária Esquerda do Tronco Pulmonar, em Evolução Natural em Adulta Assintomática de 75 Anos

**DOI:** 10.36660/abc.2019-0486

**Published:** 2020-05-11

**Authors:** Edmar Atik, Oliver Kligerman, Luiz Kajita

**Affiliations:** Hospital das Clínicas Faculdade de Medicina Universidade de São Paulo São PauloSP Brasil Instituto do Coração do Hospital das Clínicas da Faculdade de Medicina da Universidade de São Paulo, São Paulo, SP – Brasil

**Keywords:** Cardiopatias Congênitas, Síndrome de Bland White-Garland, Anormalidades dos Vasos Coronários/ diagnóstico por imagem, Isquemia

## Dados clínicos

Paciente evolui sem sintomas nos afazeres habituais como doméstica. Nega qualquer sintoma como palpitações, dor precordial ou cansaço. Cateterismo cardíaco revelou o diagnóstico de origem anômala da artéria coronária esquerda (CE) do tronco pulmonar (TP) após teste ergométrico alterado em exame de rotina, com 62 anos de idade. Desde então, refere a continuidade de seu bem-estar, mesmo após o conhecimento da existência da sua anomalia. Em uso de rosuvastatina, levotiroxina e vitamina D.

Exame físico: Bom estado geral, eupneica, acianótica, pulsos normais nos 4 membros. Peso: 49,8 Kg, Alt.: 143 cm, PAMSD:120 x 80 mmHg, FC: 74 bpm.

Precórdio: *Ictus cordis* desviado da linha hemiclavicular esquerda e algo impulsivo, e sem impulsões sistólicas na borda esternal esquerda. Bulhas cardíacas hiperfonéticas, estando a segunda bulha desdobrada. Sopro contínuo +/++/4, suave, mais audível na fúrcula e no 1^o^ e 2^o^ espaços intercostais à esquerda. Fígado não palpado e pulmões limpos.

## Exames complementares

**Figura 1:**
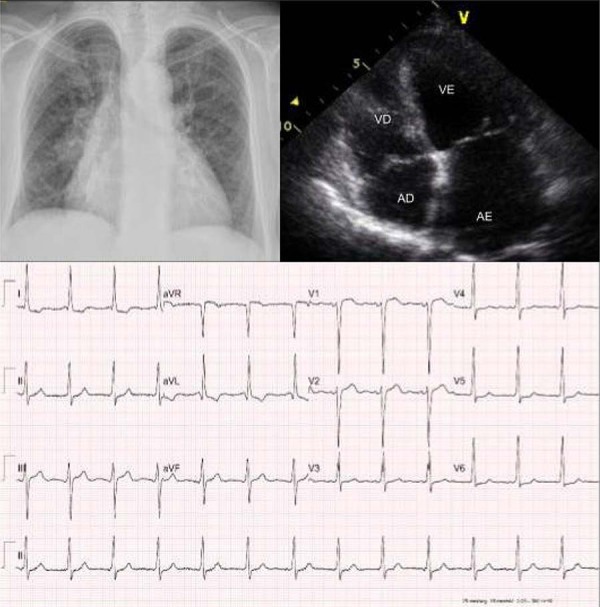
- Radiografia de tórax salienta a área cardíaca e trama vascular pulmonar aumentadas. Eletrocardiograma com sobrecarga das cavidades esquerdas com isquemia de parede ântero-lateral esquerda. Ecocardiograma com projeção em 4 câmaras salienta o aumento das cavidades esquerdas. AD: átrio direito; AE: átrio esquerdo; VD: ventrículo direito; VE: ventrículo esquerdo.

**Eletrocardiograma (ECG):** Ritmo juncional, com onda P achatada no plano frontal e nas precordiais esquerdas. Onda T negativa em I, L e de baixa amplitude de V4 a V6, indicativa de isquemia ântero-lateral. Sinais de sobrecarga das cavidades esquerdas com onda P +- em V1 e índice de Sokolof de 37 mm. QRS de 102 ms (AQRS= 0º, AT= +110º) ( Figura 1 ).

**Radiografia de tórax:** Aumento moderado da área cardíaca à custa do arco ventricular esquerdo alongado (ICT=0,68). Trama vascular pulmonar nitidamente aumentada ( Figura 1 ).

**Ecocardiograma** : Conexão atrioventricular e ventrículo-arterial normais. Aumento importante do átrio esquerdo (52 mm com volume = 125 ml/m^2^), estando normais as demais cavidades (VD= 20, VE= 56, Ao= 31), assim como as válvulas cardíacas. Não havia hipertrofia miocárdica com septo e parede posterior= 8 mm. A pressão sistólica da artéria pulmonar foi estimada pelo *Doppler* em 82 mmHg. A função biventricular era normal e a fração de ejeção do ventrículo esquerdo de 60% ( Figura 1 ).

**Figura 2:**
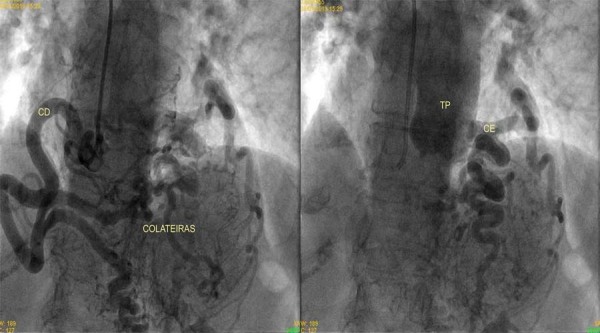
- Cinecoronariografia salienta a partir da artéria coronária direita (CD) o enchimento da artéria coronária esquerda (CE) e do tronco pulmonar (TP) na caracterização da anomalia coronária. As artérias são dilatadas e muito tortuosas, com inúmeras colaterais e sem obstruções.

**Cinecoronariografia e cateterismo cardíaco:** Artéria coronária direita (CD) de grande calibre sem obstruções e muito tortuosa. A CE, também muito tortuosa se enchia retrogradamente por circulação colateral exuberante a partir da CD. O tronco da CE desembocava no início do TP dilatado, com fluxo proveniente desde a CD. Ventriculografia esquerda mostrava contratilidade miocárdica preservada ( Figura 2 ).

**Diagnóstico clínico:** Origem anômala da artéria CE do TP, em evolução natural prolongada até 75 anos, em paciente assintomática, mas com sinais de isquemia miocárdica e de sobrecarga das cavidades esquerdas e com preservação da função miocárdica.

**Raciocínio clínico:** Havia elementos clínicos de orientação diagnóstica da cardiopatia congênita, apesar da ausência de sintomas evidentes. O sopro contínuo nítido em posição alta na fúrcula e nos espaços mais altos da borda esternal esquerda, com aumento da trama vascular pulmonar e consequentemente do átrio esquerdo, aliado a fenômenos isquêmicos no ECG orientariam para o diagnóstico da origem da artéria CE do TP. Esse diagnóstico clínico elaborado não pôde ser realizado anteriormente pela falta de sintomas, mas também pela negligência de um exame clínico semiológico devidamente realizado e avaliado, com a acurácia adequada. O diagnóstico no caso foi estabelecido pelo cateterismo cardíaco.

**Diagnóstico diferencial:** Outras cardiopatias que se acompanham de sopro contínuo correspondem a persistência do canal arterial, janela aortopulmonar e fístulas arteriovenosas em geral. No entanto, os sinais de isquemia miocárdica descritos e evidentes pelo ECG e teste ergométrico alterado não ocorrem nestas outras anomalias referidas, a não ser que haja obstrução coronária por aterosclerose associada.

**Conduta:** Em face da repercussão de volume das artérias pulmonares com hiperfluxo, e também para as cavidades esquerdas e ainda com isquemia miocárdica, pensou-se na possibilidade da eliminação da junção da artéria CE com o TP através da simples ligadura terminal da artéria coronária. Com esse pensamento haveria principalmente a preservação da função ventricular, além da eliminação da sobrecarga de volume das cavidades esquerdas, na profilaxia de fenômenos adversos a maior prazo adiante. No entanto, como a paciente se mostra sem sintomas e segundo ela própria, como seu quadro pouco se modificou desde sua descoberta há cerca de13 anos, optou-se pela conduta expectante. A recusa à operação também se sucedeu em casos semelhantes relatados na literatura.^1 - 3^

**Comentários:** A evolução natural desta paciente até a terceira idade avançada e sem sintomas e com poucas manifestações desfavoráveis, é sem dúvida um fenômeno muito raro. Essa evolução até favorável e em boas condições clínicas e hemodinâmicas se deveu primeiro à exuberante circulação colateral a partir da artéria CD podendo suprir adequadamente a circulação coronária como um todo. A isquemia ântero-lateral no ECG não se expressou com outros elementos em desacerto que causassem problemas à paciente. Espera-se daqui em diante o aparecimento de arritmias, disfunção miocárdica e até fenômenos de trombose e de embolia. Esses caracteres adquiridos que interferem na evolução a mais tempo decorrido deveriam já terem se manifestado anteriormente. Cerca de 90% desses pacientes falecem nos primeiros anos de idade, caso não sejam corrigidos cirurgicamente, e muito poucos alcançam maior idade.^1 - 3^ Ressalte-se que a intervenção operatória, mesmo na idade adulta se mostra favorável com regressão da sobrecarga de volume e de fenômenos de isquemia, como se sucedeu com 50 pacientes relatados, em idades de 31,6+ 15,6.^4^
