# Supplementary material for: Caso 2/2020 – Origem Anômala da Artéria Coronária Esquerda do Tronco Pulmonar, em Evolução Natural em Adulta Assintomática de 75 Anos
Source: Arq Bras Cardiol. 2020 May 11;114(4 Suppl 1):40–2. [Article in Portuguese] doi: 10.36660/abc.2019-0486 (PMC8149118; doi:10.36660/abc.2019-0486)
Supplement: Translation

# Translation: Case 2/2020 – Anomalous Origin of the Left Coronary Artery from the Pulmonary Trunk, Under Natural Evolution in a 75-Year-old Asymptomatic Woman

**Authors:** Edmar Atik, Oliver Kligerman, Luiz Kajita

**Affiliations:** Brazil Instituto do Coração do Hospital das Clínicas da Faculdade de Medicina da Universidade de São Paulo, São Paulo, SP – Brazil

**Keywords:** Congenital Heart Disease, Bland White-Garland Syndrome, Coronary Vessel Anomalies/diagnostic imaging, Ischemia

## Clinical data

Patient evolves without symptoms in her daily routine as a domestic worker. She denies any symptoms of palpitations, precordial pain or fatigue. When she was aged 62, a cardiac catheterization revealed the diagnosis of anomalous origin of the left coronary artery from the pulmonary artery (ALCAPA, or Bland-White-Garland syndrome), after an altered routine exercise stress test. Since then, the patient reports her well-being, although she is aware of the existence of this anomaly. She is being treated with rosuvastatin, levothyroxine and vitamin D.

Physical examination: good overall status, eupneic, acyanotic, normal pulses in the four limbs. Weight: 49.8 Kg, Height: 143 cm, BP:120 x 80 mmHg, HR: 74 bpm.

Precordium: Patient presented ictus cordis impulsive and displaced to the left of the midclavicular line, without systolic impulses at left sternal border. Hyperphonetic heart sound, with a split S2. Mild continuous murmur, +/++/4, more intense in the suprasternal notch and in the 1st and 2nd left intercostal spaces. Non-palpable liver and clean lungs.

## Complementary examinations

**Electrocardiography (ECG):** Junctional rhythm, with a flat P wave in the frontal plane and left precordial leads. Negative T waves in leads I, L, and of low amplitude in leads V4-V6, suggestive of anterolateral ischemia. Signs of overload of left cavities with a biphasic P wave in lead V1 and a Sokolof index equal to 37mm. QRS 102 ms (AQRS= 0º, AT= +110º) ( Figure 1 ).

**Figure 1:** - Chest x-ray highlights increased cardiac area and pulmonary vascular network. ECG shows overload of left cavities with inferolateral wall ischemia of the left ventricle. A 4-chamber view echocardiogram study highlights increased left cavities. RA: right atrium; LA: left atrium; RV: right ventricle; LV: left ventricle.

**Chest radiography:** Slightly enlarged cardiac area, with elongated left ventricular arch (CTI=0.68). Clearly increased pulmonary vascular network ( Figure 1 ).

**Echocardiography** : Normal atrioventricular and ventriculoarterial connections. Significantly increased left atrium size (52 mm; LAVI = 125 ml/m^2^). The other cavities (RV= 20, LV= 56, RA= 31), as well as the cardiac valves, were normal. There was no myocardial hypertrophy, with septum and posterior wall = 8 mm. The pulmonary artery systolic pressure was 82 mmHg as estimated by Doppler echocardiography. Biventricular function was normal and left ventricular ejection fraction was 60% ( Figure 1 ).

**Cinecoronariography and cardiac catheterization:** Large and extremely tortuous right coronary (RC) artery without obstruction. There was retrograde filling of the left coronary artery, which was also quite tortuous, by exuberant collateral circulation from the RC. The LC artery trunk flowed at the beginning of the dilated pulmonary trunk, with the flow originating from the RC. Left ventriculography showed preserved myocardial contractility ( Figure 2 ).

**Figure 2:** - Cinecoronariography demonstrates filling of left coronary (LC) artery and pulmonary artery trunk (PAT) from the right coronary (RC) artery, a characterization of coronary artery anomalies. The arteries are dilated and extremely tortuous, with several collateral pathways and no obstruction.

**Clinical Diagnosis:** Anomalous origin of the left coronary artery from the pulmonary artery (ALCAPA), under prolonged natural evolution in asymptomatic patient until 75 years of age, but with signs of myocardial ischemia, left cavities overload and preserved myocardial function.

**Clinical Reasoning:** There were clinical elements of diagnostic orientation of congenital heart disease, despite the absence of clear symptoms. A clear continuous murmur located at the suprasternal notch and at the uppermost spaces of the left sternal border, with an increase in pulmonary vascular network leading to an increase in left atrium, in combination with ischemic events, as detected by the ECG, would orient towards the diagnosis of anomalous origin of the LCA from the PA trunk. This elaborated clinical diagnosis could not be made earlier due to the lack of symptoms, but also to the lack of a duly performed and evaluated symptomatic/clinical examination, with adequate accuracy. The diagnosis in this case was established by the cardiac catheterization.

**Differential diagnosis:** Other heart diseases that combine with continuous murmur refer to persistent arterial channel, aortopulmonary window and arteriovenous fistulas in general. However, the signs of myocardial ischemia described and evident at the ECG, in combination with altered stress testing, do not occur in the other anomalies just mentioned, unless there is a blockage of the coronary arteries caused by atherosclerosis.

**Approach:** In face of the impact of hyperflow on the pulmonary circulation, and also on left heart cavities, in addition to myocardial ischemia, we considered the possibility of removing the junction point of the left coronary artery and the pulmonary trunk by means of a simple terminal ligature of the coronary artery. With this in view, we would mainly preserve the ventricular function, as well as eliminate left ventricular volume overload. As a consequence, we would prevent long-term adverse events. Nevertheless, since the patient presents asymptomatic and – according to herself – her clinical picture has changed very little since her diagnosis, around 13 years ago, we decided to adopt an expectant strategy. Refusal to surgery has also occurred in similar cases reported in the literature.^1 - 3^

**Comments:** This patient’s natural evolution until advanced age, with no symptoms and a few adverse manifestations, is undoubtedly a very rare phenomenon. This favorable evolution, under good clinical and hemodynamic conditions, was primarily due to the exuberant collateral circulation from the RC, which managed to ensure adequate coronary circulation as a whole. The anterolateral ischemia revealed by the ECG was not combined with other elements that could be harmful to the patient. From now on, there may be the new onset of arrhythmias, myocardial dysfunction and even thrombosis and embolism events. These acquired traits, which have an effect on the evolution, should have manifested earlier. About 90% of these patients die within the first years of life, if they are not surgically treated, and very few reach higher age.^1 - 3^ It is important to highlight that these patients can benefit from surgical treatment even in adulthood, leading to the decrease of volume overload and of ischemic events, as in a study reported elsewhere with a sample of 50 patients, with an average age of 31.6 ± 15.6 years.^4^
